# Insights on United States cumulative measles cases during the 2026 outbreak using ARIMA and generalized linear models

**DOI:** 10.3389/fepid.2026.1921780

**Published:** 2026-09-03

**Authors:** Antonio Gondim, Chidozie W. Chukwu, Folashade B. Agusto, Joshua Ologbonyo

**Affiliations:** 1 Mathematics, University of Miami, Coral Gables, FL, United States; 2 Mathematical Sciences, Georgia Southern University, Statesboro, GA, United States; 3 Ecology and Evolutionary Biology, University of Kansas, Lawrence, KS, United States; 4 Mathematics and Applied Mathematics, North-West University, Potchefstroom, South Africa; 5 Mathematics and Statistics, Mercer University, Macon, GA, United States

**Keywords:** ARIMAX, measles, negative binomial regression, outbreak prediction, poisson regression, public health, time series analysis

## Abstract

To understand the nature of the current measles outbreaks taking place across the United States in 2026, we analyzed historical surveillance data dating back to 1997 using four distinct forecasting models. By withholding data from the first four months of 2026 for out-of-sample validation, we evaluated how well traditional trend tracking and temporal dependence could anticipate this year's trajectory. While models incorporating short-term patterns performed best overall, every single model underestimated the cumulative cases recorded by May 1, 2026. This systematic underprediction reveals that the ongoing resurgence cannot be explained merely as a continuation of past trends or 2025 outbreak effects. Instead, 2026 represents a distinct, more severe outbreak regime, as the projected annual burden was already reached by May. These findings indicate that public health tracking must adapt to this heightened risk environment, and warn that the final 2026 case burden will substantially exceed that of previous years.

## 1 Introduction

Measles remains one of the most contagious vaccine-preventable infectious diseases worldwide despite the availability of a highly effective vaccine. Although endemic measles transmission was declared eliminated in the United States in 2000, imported infections and subsequent outbreaks continue to occur, particularly in communities with suboptimal vaccination coverage (1, 2). Over the post-elimination period, the U.S. measles time series is characterized by generally low annual incidence punctuated by intermittent outbreak years, often driven by importations and clusters of under-vaccinated populations (1, 2). Notable resurgences in recent years, including outbreaks in 2014, 2019, 2025, and 2026, have raised concerns about declining vaccination coverage, increased international travel, vaccine hesitancy, and the accumulation of susceptible individuals within certain communities.

Accurate forecasting of measles incidence is critical for public health preparedness and resource allocation. Reliable forecasts can provide early warning signals for potential outbreaks, support surveillance systems, and inform targeted vaccination and containment strategies. Given the increasing irregularity of measles outbreaks and their dependence on both epidemiological and social drivers, there is a growing need for statistical approaches capable of capturing temporal dynamics and abrupt changes in transmission patterns.

Traditional infectious disease forecasting approaches include mechanistic compartmental epidemic models, generalized linear models, and time-series methods such as autoregressive integrated moving average (ARIMA) models. While mechanistic models provide insight into transmission processes, statistical forecasting models are often more suitable for short-term prediction when surveillance data are readily available and when underlying transmission mechanisms are difficult to quantify precisely.

Count-regression models are particularly natural for infectious disease surveillance data because reported cases are nonnegative integers. Poisson generalized linear models (GLMs) provide a standard baseline framework for modeling such count data and interpreting multiplicative effects of covariates (3). However, infectious disease data frequently exhibit overdispersion, for which negative binomial regression provides a flexible extension (4). These models and their variants have been widely used in outbreak detection and surveillance contexts, including Poisson- and negative binomial-based approaches for monitoring deviations from expected incidence patterns (5–7).

### 1.1 Existing literature

Forecasting infectious disease incidence has become an important component of modern public health surveillance. Numerous statistical and mathematical approaches have been developed to predict disease burden, assess outbreak risk, and support intervention planning. For measles specifically, forecasting efforts have ranged from mechanistic transmission models to purely statistical time-series methods.

Early studies focused primarily on compartmental epidemic models that incorporated susceptible, exposed, infectious, and recovered populations to investigate measles transmission dynamics and vaccination strategies. Anderson and May (8) demonstrated the importance of herd immunity thresholds and vaccination coverage in determining long-term measles persistence. Subsequent studies extended these approaches by incorporating age structure, seasonality, spatial heterogeneity, and stochastic transmission effects.

In North America, several investigations have examined measles resurgence following the elimination era. Fiebelkorn et al. (2) analyzed post-elimination measles outbreaks in the United States and emphasized the role of imported infections and under-vaccinated communities. Patel et al. (9) documented the largest U.S. measles outbreak since 1992 and highlighted the vulnerability of communities with declining vaccine uptake. More recent reports from the Centers for Disease Control and Prevention (CDC) have documented substantial increases in measles incidence during 2025 and 2026, raising renewed concerns regarding outbreak persistence and the erosion of herd immunity (10).

From a forecasting perspective, generalized linear models have been widely used for infectious disease surveillance because disease counts are naturally represented as nonnegative integer-valued outcomes (3). Poisson regression remains a common baseline approach for modeling expected disease counts and assessing the influence of explanatory variables. However, infectious disease data frequently exhibit overdispersion, leading researchers to adopt Negative Binomial models that provide greater flexibility in variance modeling (4). Negative Binomial regression has been shown to outperform Poisson regression in many epidemiological applications where disease incidence exhibits substantial variability and outbreak-driven spikes. ARIMA and SARIMA models have also been applied specifically to measles incidence forecasting from national surveillance data (11).

Time-series forecasting methods have also been extensively applied to infectious disease surveillance. ARIMA and ARIMAX models have been successfully used to forecast influenza, dengue fever, COVID-19, measles, and other communicable diseases (12–15). These models account for temporal dependence and can incorporate exogenous predictors representing environmental, demographic, or outbreak-related factors. Such approaches often provide competitive short-term forecasting performance when strong serial correlation exists within surveillance data.

More recently, machine learning and hybrid forecasting frameworks have been applied to infectious disease prediction. Methods such as artificial neural networks, long short-term memory (LSTM) networks, gated recurrent units (GRUs), Prophet models, and hybrid ARIMA-machine learning frameworks have demonstrated improved predictive capabilities in several infectious disease settings. Nevertheless, these approaches often sacrifice interpretability, which remains an important consideration for public health decision-making.

Despite the growing literature on infectious disease forecasting, relatively few studies have focused on forecasting cumulative measles cases in the post-elimination United States. While annual incidence forecasting provides valuable information for short-term outbreak prediction and surveillance planning, cumulative disease burden offers a more comprehensive measure of the overall public health impact of measles by capturing the total number of infections accumulated over time. This perspective is particularly important for measles, where transmission dynamics are influenced by vaccination gaps, immunity levels, and intermittent outbreak events. Therefore, evaluating cumulative burden allows for a broader assessment of long-term disease consequences and the effectiveness of control strategies beyond year-to-year fluctuations in reported cases. Existing studies generally emphasize outbreak descriptions, vaccination coverage, or mechanistic transmission dynamics rather than statistical forecasting of long-term cumulative burden. Furthermore, limited attention has been given to comparing count-regression models with time-series approaches while explicitly incorporating major outbreak-related structural shifts. This gap motivates the present study.

### 1.2 Motivation and research objectives

The resurgence of measles in the United States in recent years highlights the need for reliable forecasting tools capable of anticipating future disease burden. Although measles was declared eliminated in the United States in 2000, repeated outbreaks have demonstrated that elimination does not guarantee permanent protection against resurgence. Declining vaccination coverage, vaccine hesitancy, international importations, and localized clusters of susceptible individuals continue to create conditions conducive to outbreaks.

The primary motivation of this study is to develop and evaluate statistically rigorous forecasting models capable of quantifying and predicting future measles burden in the United States using publicly available surveillance data. Unlike annual incidence forecasting, which focuses primarily on short-term fluctuations, cumulative case forecasting provides a broader measure of long-term disease burden and captures the persistent effects of major outbreaks on national measles trends.

To address this need, we compare several statistical forecasting approaches, including Poisson generalized linear models (GLMs), quasi-Poisson GLMs, negative binomial GLMs, and an ARIMAX grid-search model. These methodologies offer complementary advantages for modeling count data, accommodating departures from standard Poisson assumptions, accounting for temporal dependence, and incorporating outbreak-related structural changes in disease burden.

The specific objectives of this study are:

1. To develop forecasting models for cumulative U.S. measles cases using Poisson GLM, quasi-Poisson GLM, negative binomial GLM, and an ARIMAX grid-search methodology.
2. To quantify the effects of major outbreak periods, including the 2014, 2019, and 2025 measles resurgences, on long-term cumulative disease burden.
3. To evaluate and compare count-regression and time-series forecasting approaches using in-sample error measures, the held-out 2026 forecast comparison, and rolling one-year-ahead validation over the 2016–2025 test period.
4. To assess the predictive performance of competing forecasting frameworks and identify models that balance interpretability, statistical rigor, and forecasting accuracy.
5. To provide evidence that can support disease surveillance, vaccination strategies, outbreak preparedness, and public-health decision making in the United States.

To the best of our knowledge, this study is among the first to systematically compare count-regression and time-series forecasting approaches for cumulative measles burden in the post-elimination United States while explicitly accounting for outbreak-related structural shifts in the cumulative disease trajectory. In this study, we investigate the forecasting of cumulative measles burden in the United States using both count-regression and time-series approaches. Specifically, we compare Poisson generalized linear models (GLMs), quasi-Poisson GLMs, negative binomial GLMs, and an ARIMAX grid-search model that incorporates outbreak-related covariates. The count-regression models are designed to capture persistent shifts in cumulative disease burden associated with major outbreak periods, while the ARIMAX grid-search framework additionally accounts for temporal dependence in the surveillance series. By evaluating model performance using in-sample error measures, including mean squared error (MSE) and root mean squared error (RMSE), together with the held-out 2026 forecast comparison and rolling one-year-ahead validation over the 2016–2025 test period, we aim to identify forecasting approaches that provide reliable short-term predictions while maintaining statistical interpretability and relevance for public-health surveillance and decision-making. The present analysis focuses on cumulative measles cases. Let $\left(C_{y}\right)$ denote the number of reported measles cases in year (y), and define

$$Y_{y}=\sum_{\;j\leq y}C_{j}$$

as the cumulative number of cases through year (y). Because the cumulative outcome permanently retains cases from previous years, major outbreaks produce lasting upward shifts in the cumulative trajectory. This characteristic motivates the inclusion of outbreak-related level-shift indicators to capture the persistent impact of major resurgence periods on cumulative measles burden.

### 1.3 Road map

The remainder of this paper is organized as follows. Section 2.1 describes the U.S. measles surveillance data, the construction of the cumulative case series, and the preprocessing procedures used to prepare the dataset for analysis. Section 2.2.1 presents the forecasting methodologies considered in this study, including the Poisson generalized linear model (GLM), quasi-Poisson GLM, negative binomial GLM, and ARIMAX grid-search model, together with the corresponding model specifications and evaluation criteria. Section 3 reports the empirical results, including parameter estimates, model diagnostics, forecasting performance measures, and the held-out validation analysis based on 2026 cumulative measles data. Comparative assessments of the competing forecasting frameworks are also presented. Section 4 discusses the epidemiological and statistical implications of the findings, highlights the strengths and limitations of the proposed approaches, compares the results with existing studies, and outlines directions for future research. Finally, Section 4.5 summarizes the main conclusions and their relevance for measles surveillance, outbreak preparedness, and public-health decision-making in the United States.

## 2 Materials and methods

### 2.1 Data and time indexing

The annual case series is first sorted by count year and then transformed into cumulative cases. Although the cumulative series is computed from the full available annual record, the forecasting models are trained on observations beginning in the 1996 reporting year. The 2026 observation is held out and forecasted separately. The cumulative value for count year y is plotted at January 1 of year (y+1). Therefore, a point shown at January 1, 2020 corresponds to the cumulative total through the 2019 count year. This indexing convention is used only for visualization and reporting. All covariates are defined using the original epidemiological count year. For example, the 2019 outbreak indicator is activated according to y≥2019, even though the cumulative value including 2019 cases is displayed at January 1, 2020.

#### 2.1.1 Data and extraction

**Figure 1:**
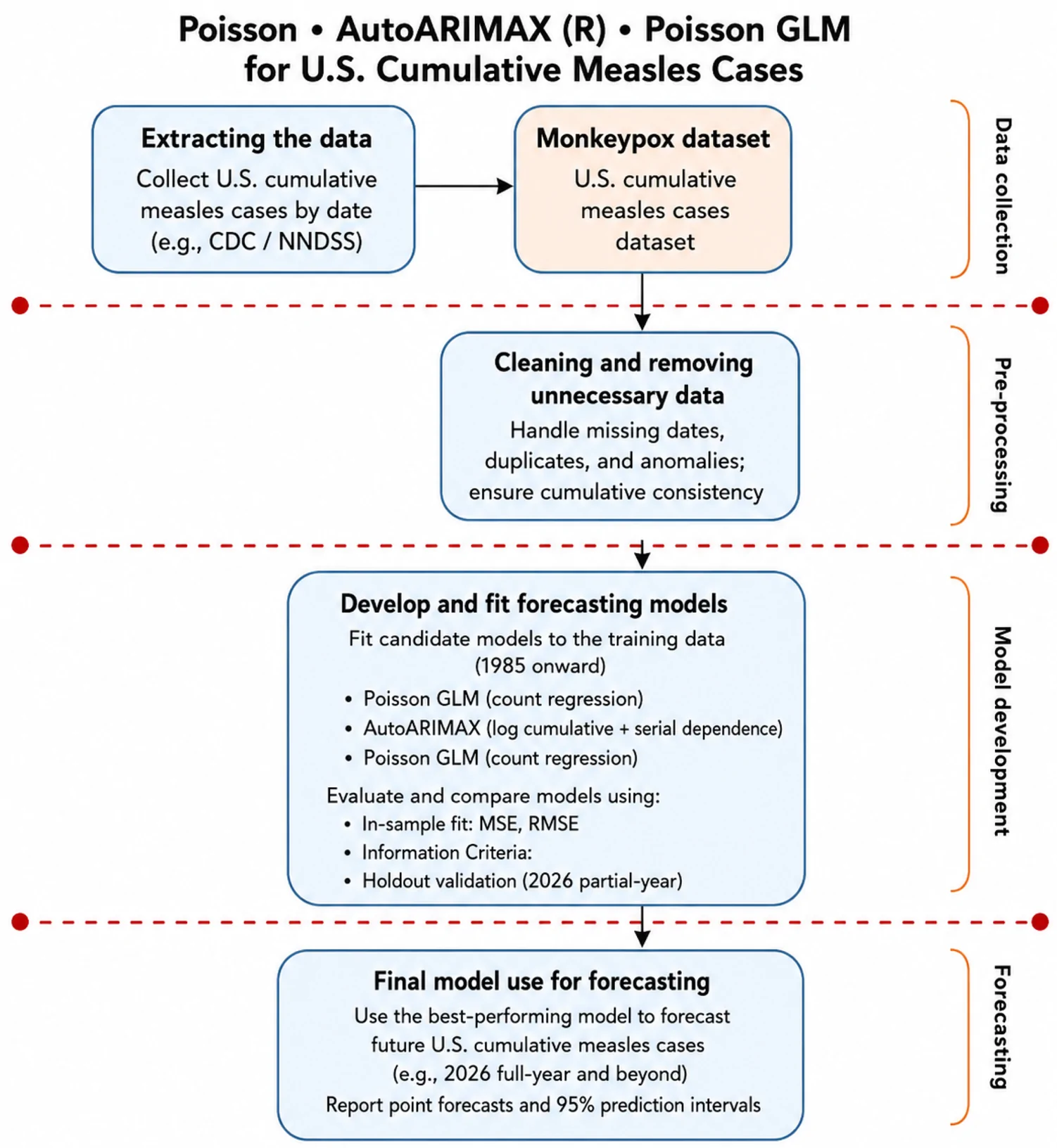
Workflow for data collection, preprocessing, model development, validation, and forecasting of cumulative U.S. measles cases. Model performance is assessed using MSE, RMSE, the held-out 2026 comparison, and rolling one-year-ahead validation over the 2016–2025 test period before generating forecasts and prediction intervals.

Figure 1 illustrates the end-to-end workflow for processing, modeling, and forecasting cumulative measles cases in the United States. The process begins with data collection, where annual cumulative measles surveillance records are obtained from publicly available sources (16) and organized into a structured time-series dataset. The collected data then undergo preprocessing, including the identification and treatment of missing observations, removal of duplicate records, correction of inconsistencies, and verification of cumulative consistency across years. The 2026 validation dataset consists of cumulative measles cases reported through May 1, 2026, obtained from the CDC Measles Cases and Outbreaks surveillance data [accessed via the Beacon reporting platform (17)]. Because these surveillance data are continuously updated as case investigations are completed, the reported cumulative total should be regarded as provisional rather than final. Consequently, the out-of-sample validation presented in this study is based on the best available surveillance data at the time of analysis, and minor revisions to the reported case counts may occur in subsequent CDC updates. However, such revisions are not expected to materially affect the qualitative conclusions regarding the comparative forecasting performance of the models. Following preprocessing, the cleaned dataset is used for model development and evaluation. Multiple forecasting approaches are fitted to the historical cumulative measles series, including Poisson generalized linear models (GLMs), quasi-Poisson GLMs, negative binomial GLMs, and an ARIMAX grid-search model. The count-regression models incorporate outbreak-related covariates to capture persistent shifts in cumulative disease burden, while the ARIMAX grid-search model combines outbreak indicators with temporal dependence. Model performance is evaluated using in-sample forecasting metrics, including the mean squared error (MSE) and root mean squared error (RMSE), together with an out-of-sample validation exercise in which the 2026 observation is withheld from model fitting and used as a holdout forecast target. To provide broader evidence of short-term forecast performance, the models are also evaluated using rolling one-year-ahead validation over the 2016–2025 test period.

The final stage of the workflow consists of forecasting future cumulative measles burden using the best-performing model. Forecasts are accompanied by 95% prediction intervals to quantify uncertainty and provide insight into the potential range of future disease burden. This framework enables systematic comparison of alternative forecasting approaches and supports epidemiological surveillance, outbreak monitoring, and evidence-based public health decision-making.

#### 2.1.2 Rationale for model selection

The forecasting models considered in this study were selected for both methodological and practical reasons. First, all four frameworks provide formal uncertainty quantification through confidence intervals or prediction intervals, which is essential for public-health decision making where uncertainty must be explicitly communicated. Reliable uncertainty estimates enable policymakers and public-health agencies to assess potential risks and prepare for a range of outbreak scenarios rather than relying solely on point forecasts. Second, the selected methods are well-suited to annual surveillance datasets such as the one analyzed in this study. Because annual measles surveillance data contain a relatively small number of observations, highly parameterized machine-learning approaches may be difficult to estimate reliably and may suffer from overfitting. In contrast, the selected statistical models can be estimated efficiently while maintaining interpretability and statistical rigor. Third, the models represent complementary forecasting philosophies. The Poisson, quasi-Poisson, and negative binomial models emphasize count-data characteristics and the estimation of outbreak-related effects, providing interpretable measures of how major resurgence periods influence cumulative disease burden. The ARIMAX grid-search model, on the other hand, focuses on exploiting temporal dependence and recent observations to improve predictive performance. Comparing these approaches, therefore, provides insight into the relative importance of outbreak-related covariates and serial dependence in forecasting cumulative measles burden. Lastly, the selected models have been widely applied in epidemiology, public health surveillance, and infectious disease forecasting. Their established theoretical foundations, interpretability, and ability to generate uncertainty estimates make them attractive tools for disease surveillance applications. By comparing these frameworks within a common forecasting setting, this study provides a practical assessment of their suitability for forecasting cumulative measles burden in the post-elimination United States.

### 2.2 Model analytical methods

#### 2.2.1 Count-regression model formulation

We fit three generalized count-regression models to the cumulative outcome $Y_{y}$: Poisson, quasi-Poisson, and negative binomial. All three models use the same log-linear mean structure,

$$E(Y_{y})=\lambda_{y},\;\log(\lambda_{y})=\eta_{y},$$

where

$$\eta_{y}=\alpha +\gamma_{\mathrm{time}}T_{y}+\gamma_{2014}O_{2014,y}+\gamma_{2019}O_{2019,y}+\gamma_{2025}O_{2025,y}.$$

Equivalently,

$$\lambda_{y}=\exp\left(\alpha +\gamma_{\mathrm{time}}T_{y}+\gamma_{2014}O_{2014,y}+\gamma_{2019}O_{2019,y}+\gamma_{2025}O_{2025,y}\right).$$

Here,

$$T_{y}=y-1996$$

is the baseline cumulative time trend. The outbreak indicators are cumulative level-shift indicators:

$$O_{2014,y}=\left\{ \begin{array}{cc} 1,\; & y\geq 2014,\;\\ 0,\; & y<2014,\; \end{array}\right.\;O_{2019,y}=\left\{ \begin{array}{cc} 1,\; & y\geq 2019,\;\\ 0,\; & y<2019,\; \end{array}\right.$$

and

$$O_{2025,y}=\left\{ \begin{array}{cc} 1,\; & y\geq 2025,\;\\ 0,\; & y<2025.\; \end{array}\right.$$

These variables do not represent one-year outbreak pulses. Instead, they reflect the fact that once cases occur during an outbreak year, they remain part of the cumulative total in all later observations. The 2019 indicator is motivated by the large 2019 U.S. measles resurgence, during which CDC reported the highest annual case count since 1992 (9). The 2025 indicator is motivated by the large outbreak activity reported by CDC in 2025, including outbreak-associated cases in New Mexico, Oklahoma, and Texas (10, 16).

The three count-regression models differ only in their variance assumptions. The Poisson model assumes

$$Y_{y}∼\text{Poisson}(\lambda_{y}),\;\text{Var}(Y_{y})=\lambda_{y}.$$

The quasi-Poisson model keeps the same mean structure but allows the variance to be scaled by an estimated dispersion parameter ϕ:

$$\text{Var}(Y_{y})=\phi \lambda_{y}.$$

When ϕ>1, the quasi-Poisson model allows overdispersion relative to the Poisson model. When ϕ<1, it indicates underdispersion relative to the Poisson variance assumption.

The negative binomial model also keeps the same log-linear mean structure but uses a quadratic mean-variance relationship:

$$Y_{y}∼\text{Negative Binomial}(\lambda_{y},\kappa ),\;\text{Var}(Y_{y})=\lambda_{y}+\kappa \lambda_{y}^{2},$$

where κ is the negative binomial dispersion parameter. When κ is close to zero, the negative binomial model approaches the Poisson variance structure.

Additional covariates were considered during model development. A post-elimination indicator was excluded from the final count-regression specification because the training set begins in 1996, leaving only a small number of observations before the 2,000 elimination milestone. A COVID-period indicator was also evaluated but removed because it was not statistically distinguishable from zero once the baseline time trend and outbreak shifts were included.

#### 2.2.2 ARIMAX grid-search pipeline and model definition

As a fourth forecasting approach, we fit a ARIMAX model with external regressors. In response to reviewer concerns about the previous AutoARIMAX specification, we no longer combine first differencing with permanent post-outbreak level-shift indicators. Instead, annual measles counts are modeled on the $\log(1+C_{y})$ scale using d=0, an explicit linear time trend, and a one-year outbreak pulse indicator. This follows the ARIMA modeling framework for time series with external regressors (12–14, 18).

The data-processing pipeline for constructing the cumulative case series and assigning plotting dates is the same as for the count-regression models. Annual cases are sorted by count year, cumulative cases are computed for reporting purposes, and the plotting date is assigned as January 1 of the following year. For count year 2026, the observed partial-year cumulative value is indexed at May 1, 2026. The ARIMAX grid-search model is trained on count years 1996–2025, and the 2026 full-year cumulative forecast is displayed at December 31, 2026.

Let $C_{y}$ denote annual measles cases in count year y. The modeled response is

$$Z_{y}=\log(1+C_{y}).$$

The ARIMAX model combines a regression mean with an autoregressive error process. Specifically,

$$Z_{y}=\beta_{1}\;{\text{time}}_{y}+\beta_{2}\;\text{major}\_{\text{outbreak}}_{y}+u_{y},$$

where $u_{y}$ is the serially correlated regression error,

$${\text{time}}_{y}=y-1996,$$

and

$$\text{major}\_{\text{outbreak}}_{y}=1\{y\in \{2014,2025\}\}.$$

The outbreak term is therefore a one-year pulse indicator, not a permanent post-outbreak level shift, which addresses the redundancy between differencing and level-shift indicators identified in the previous specification. We retained only the 2014 and 2025 pulses in the ARIMAX model. Although the count-regression models use a separate 2019 outbreak-shift indicator, adding a distinct 2019 pulse to the ARIMAX specification led to parameter-identifiability problems: with only annual observations and a short series, the additional pulse was weakly identified and highly collinear with the autoregressive dynamics and the estimated time trend, producing unstable coefficients and inflated standard errors. We therefore adopted the more parsimonious two-pulse specification, in which the 2019 resurgence is absorbed by the autoregressive error process rather than by a dedicated indicator.

Candidate models were evaluated using an explicit grid search over ARIMAX(k,0,0) and ARIMAX(n,0,m) specifications, rather than automatic stepwise order selection. The final selected model was ARIMAX(2,0,0), so the selected error process is

$$u_{y}=\phi_{1}u_{y-1}+\phi_{2}u_{y-2}+\varepsilon_{y},$$

where $\varepsilon_{y}$ is a white-noise innovation with mean zero and constant variance. Equivalently, the fitted model can be written as

$$\begin{array}{rl} Z_{y} & =\beta_{1}\;{\text{time}}_{y}+\beta_{2}\;\text{major}\_{\text{outbreak}}_{y}\\ & +\phi_{1}\left(Z_{y-1}-\beta_{1}\;{\text{time}}_{y-1}-\beta_{2}\;\text{major}\_{\text{outbreak}}_{y-1}\right)\\ & +\phi_{2}\left(Z_{y-2}-\beta_{1}\;{\text{time}}_{y-2}-\beta_{2}\;\text{major}\_{\text{outbreak}}_{y-2}\right)+\varepsilon_{y} \end{array}$$

This expanded form makes explicit that the autoregressive terms are applied to the regression residuals rather than to an undefined latent process. All fitted values, forecasts, and confidence intervals were obtained on the $\log(1+C_{y})$ scale for annual cases and then reconstructed into cumulative counts by transforming back with the exponential function and accumulating the resulting annual increments.

## 3 Forecasting results

### 3.1 Parameter estimates for count-regression models

Table 1 reports the parameter estimates for the Poisson model. The retained covariates are the baseline time trend and the cumulative outbreak-shift indicators for 2014, 2019, and 2025. All retained coefficients are positive and statistically significant.

**Table 1:** Parameter estimates and uncertainty quantification for the Poisson model.

Parameter	Coef.	Std. Error	*z*-value	*p*-value	95% CI lower	95% CI upper	Exp. Coef.
Intercept	11.2342	0.0016	6989.3340	<0.001	11.2311	11.2374	75,677.7581
Time trend	0.0011	0.0002	6.8639	<0.001	0.0008	0.0014	1.0011
2014 outbreak shift	0.0111	0.0026	4.2896	<0.001	0.0060	0.0161	1.0111
2019 outbreak shift	0.0179	0.0023	7.6974	<0.001	0.0133	0.0225	1.0181
2025 outbreak shift	0.0286	0.0038	7.5161	<0.001	0.0211	0.0361	1.0290

Because the count-regression models use a log link, exponentiated coefficients are multiplicative effects. For example,

exp⁡(0.0286)≈1.0290,

so the 2025 outbreak shift corresponds to an estimated 2.90% multiplicative increase in the expected cumulative total, holding the other covariates fixed.

Table 2 reports the quasi-Poisson parameter estimates. The estimated coefficients are nearly identical to the Poisson estimates because the mean structure is the same. The estimated dispersion was 0.11, indicating underdispersion relative to the standard Poisson variance assumption for this cumulative series.

**Table 2:** Parameter estimates and uncertainty quantification for the quasi-Poisson model.

Parameter	Coef.	Std. Error	*z*-value	*p*-value	95% CI lower	95% CI upper	Exp. Coef.
Intercept	11.2342	0.0005	21,247.4599	<0.001	11.2332	11.2353	75,677.7581
Time trend	0.0011	0.0001	20.8661	<0.001	0.0010	0.0012	1.0011
2014 outbreak shift	0.0111	0.0008	13.0404	<0.001	0.0094	0.0127	1.0111
2019 outbreak shift	0.0179	0.0008	23.3999	<0.001	0.0164	0.0194	1.0181
2025 outbreak shift	0.0286	0.0013	22.8489	<0.001	0.0261	0.0311	1.0290

The estimated quasi-Poisson dispersion parameter was approximately 0.11, indicating pronounced underdispersion relative to the Poisson assumption. This is expected when the modeled outcome is a cumulative count. Cumulative measles cases form a smooth, monotonically increasing trajectory in which each annual value differs from the previous one by a comparatively small increment. Consequently, the observed year-to-year variability is far smaller than the variability implied by a Poisson distribution whose variance equals its large cumulative mean, driving the estimated dispersion below one. This underdispersion is therefore a structural consequence of modeling cumulative rather than incident counts.

Table 3 reports the negative binomial parameter estimates. The fitted coefficients are nearly identical to the Poisson and quasi-Poisson estimates. The estimated dispersion parameter was close to zero and not statistically significant, indicating that the negative binomial fit did not materially depart from the Poisson model for this cumulative series.

**Table 3:** Parameter estimates and uncertainty quantification for the negative binomial model.

Parameter	Coef.	Std. Error	*z*-value	*p*-value	95% CI lower	95% CI upper	Exp. Coef.
Intercept	11.2342	0.0016	6989.2220	<0.001	11.2311	11.2374	75677.3694
Time trend	0.0011	0.0002	6.8668	<0.001	0.0008	0.0014	1.0011
2014 outbreak shift	0.0111	0.0026	4.2917	<0.001	0.0060	0.0161	1.0111
2019 outbreak shift	0.0179	0.0023	7.6979	<0.001	0.0133	0.0225	1.0181
2025 outbreak shift	0.0286	0.0038	7.5118	<0.001	0.0211	0.0360	1.0290
Dispersion parameter	0.0000	0.0000	0.0149	0.9881	0.0000	0.0000	–

### 3.2 ARIMAX grid-search parameter estimates

The selected ARIMAX grid-search model was ARIMAX(2,0,0), with AIC equal to 88.80. Because the selected model contains two autoregressive terms, the first two observations were used to initialize the AR recursion, and in-sample fit metrics were therefore calculated after this two-year burn-in period. The in-sample mean absolute error was 341.24 cumulative cases, and the root mean squared error was 499.82 cumulative cases.

The two autoregressive terms were statistically significant, indicating residual temporal dependence after accounting for the time trend and outbreak pulse. The outbreak pulse was positive but not statistically significant (*p* = 0.31). This imprecision is a direct consequence of parameter-identifiability limitations: only two outbreak pulses (2014 and 2025) are present in a short annual series, and their effect is partially absorbed by the autoregressive terms and the time trend, so the pulse coefficient is weakly identified. The wide interval should therefore be read as evidence that the magnitude of the outbreak effect cannot be estimated precisely from these data, not as evidence that outbreaks have no effect. Unlike the previous AutoARIMAX specification, the model no longer produces implausibly large post-period coefficients. The result is summarized in Table 4.

**Table 4:** Parameter estimates for the ARIMAX grid-search model.

Parameter	Coef.	Std. Error	*z*-value	*p*-value	95% CI lower	95% CI upper
Time trend	0.2049	0.0540	3.7929	<0.001	0.0990	0.3108
Major outbreak year	2.1012	2.0635	1.0183	0.3085	−1.9432	6.1456
AR(1)	0.4136	0.1640	2.5219	0.0117	0.0922	0.7349
AR(2)	0.3854	0.1838	2.0970	0.0360	0.0252	0.7455
$\sigma^{2}$	0.9767	0.2169	4.5024	<0.001	0.5515	1.4019

Although AR(3) and AR(4) had lower AIC values, they were not retained because one or more AR coefficients were not statistically significant, and AR(4) also showed residual autocorrelation. The AR-only candidate models and corresponding selection criteria are summarized in Table 5. The selected AR-only model was ARIMAX(2, 0, 0).

**Table 5:** ARIMAX order selection for AR-only candidates.

Order	AIC	AR *p*-values	Ljung–Box *p*-value	Ljung–Box result	All AR *p*-values <0.05	Selected
(0,0,0)	133.97	–	<0.001	Autocorrelation detected	No	No
(1,0,0)	93.54	<0.001	0.329	No autocorrelation detected	Yes	No
(2,0,0)	88.80	0.0117; 0.0360	0.509	No autocorrelation detected	Yes	Yes
(3,0,0)	84.72	0.0341; 0.5461; 0.1495	0.082	No autocorrelation detected	No	No
(4,0,0)	84.39	0.1746; 0.5680; 0.2345; 0.5784	0.029	Autocorrelation detected	No	No

**Figure 2:**
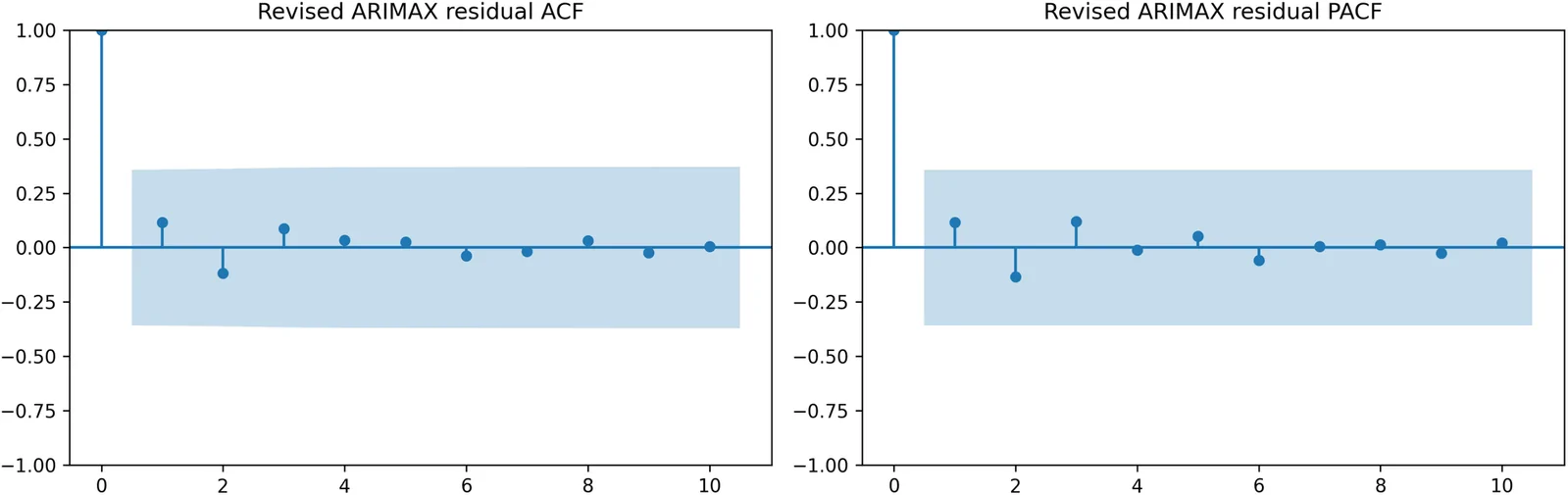
Residual ACF and PACF plots for the selected ARIMAX grid-search model.

Residual diagnostics were used to assess whether meaningful autocorrelation remained after fitting the selected ARIMAX model. As shown in Table 6, the Ljung–Box test showed no evidence of residual autocorrelation at lags 1, 5, or 10. The minimum Ljung–Box *p*-value was 0.509. Residual ACF and PACF plots are shown in Figure 2.

**Table 6:** Ljung–Box residual autocorrelation test for the selected ARIMAX model.

Lag	Ljung–Box statistic	*p*-value
1	0.4365	0.5088
5	1.2476	0.9402
10	1.3903	0.9992

We note that the equivalent residual ACF/PACF diagnostics for the Poisson, quasi-Poisson, and negative binomial models are not reported here because those model specifications were not part of the current revision; corresponding diagnostic plots for the three count-regression models can be added in a subsequent revision if required.

### 3.3 Forecast accuracy and held-out 2026 validation

The primary validation set consists of the held-out 2026 observation. As of May 1, 2026, the observed partial-year cumulative total was 84,565 cases. Since the 2026 observation was excluded from model training, this point provides a direct short-term validation target. Table 7 compares the four models using both in-sample training accuracy and held-out validation accuracy. The validation error is calculated using the raw model forecast, not the adjusted forecast. The adjusted forecast is reported only as a monotonicity correction because a full-year cumulative forecast cannot be lower than the cumulative total already observed by May 1, 2026. The count-regression models have substantially better in-sample accuracy than the ARIMAX grid-search model. The Poisson and quasi-Poisson models have training MAE = 64.60 and RMSE = 83.85, while the negative binomial model has training MAE = 64.78 and RMSE = 83.85. However, the held-out validation comparison reverses this ranking. The Poisson, quasi-Poisson, and negative binomial models underpredict the already observed 2026 cumulative total by approximately 1,723 cases, while the ARIMAX grid-search model underpredicts it by approximately 1,416 cases.

**Table 7:** Training and held-out 2026 validation accuracy across the four models.

Model	Training MAE	Training RMSE	Raw 2026 forecast	Observed 2026 cumulative	Validation absolute error	Raw implied 2026 cases
Poisson	64.60	83.85	82,841.78	84,565.00	1,723.22	90.78
Quasi-Poisson	64.60	83.85	82,841.78	84,565.00	1,723.22	90.78
Negative Binomial	64.78	83.85	82,841.77	84,565.00	1,723.23	90.77
ARIMAX grid search	341.24	499.82	83,148.71	84,565.00	1,416.29	397.71

This validation result indicates that the 2026 outbreak cannot be adequately represented by the 2025 outbreak coefficient alone. In the count-regression models, the 2025 cumulative outbreak shift produces a raw implied 2026 incidence of only about 91 cases. However, 1,814 measles cases had already been observed in 2026 by May 1. The ARIMAX grid-search model is more responsive to the recent upward movement, with a raw implied 2026 incidence of approximately 398 cases, but it still produces a raw forecast below the already observed partial-year cumulative burden. Thus, all four models point to the same epidemiological conclusion: the 2026 outbreak is not simply a continuation of the 2025 level shift and should be modeled as a separate outbreak period once sufficient data become available (see Table 8).

**Table 8:** Model summary and 2026 forecast comparison.

Quantity	Poisson	Quasi-Poisson	Negative binomial	ARIMAX grid search
Dispersion	NA	0.11	0.00	NA
Training MAE	64.60	64.60	64.78	341.24
Training RMSE	83.85	83.85	83.85	499.82
Partial 2026 observed cumulative cases	84,565.00	84,565.00	84,565.00	84,565.00
Raw forecast cumulative cases	82,841.78	82,841.78	82,841.77	83,148.71
Adjusted forecast cumulative cases	84,565.00	84,565.00	84,565.00	84,565.00
Raw forecast 2026 cases implied	90.78	90.78	90.77	397.71
Adjusted forecast 2026 cases implied	1,814.00	1,814.00	1,814.00	1,814.00
95% CI lower	82,278.67	82,656.12	82,278.66	84,565.00
95% CI upper	84,565.00	84,565.00	84,565.00	85,908.78

The 2026 hold-out comparison is based on a single partial-year observation and therefore provides limited evidence of out-of-sample performance. To address this limitation, we supplemented the 2026 hold-out comparison with rolling one-year-ahead validation over the 2016–2025 test period for all four models. In this rolling-window exercise, each test year is predicted using only observations that would have been available before that year. Specifically, for a test year y, the model is refit using data through year y−1 and then used to predict the annual increment for year y; this procedure is repeated for each year from 2016 through 2025. For the cumulative count-regression models, the predicted annual increment is obtained as the difference between the predicted cumulative total for year y and the observed cumulative total through year y−1. This design preserves the temporal ordering of the surveillance series and provides a more informative prospective validation exercise than a single 2026 hold-out point.

The rolling one-year-ahead validation results over the 2016–2025 test period are presented in Table 9. The count-regression models produced lower MAE values, whereas the ARIMAX grid-search model achieved a lower RMSE, indicating comparatively better performance in reducing larger forecast errors.

**Table 9:** Rolling one-year-ahead validation results over the 2016–2025 test period across all four models.

Model	Test years	n scored	MAE	RMSE
Poisson	2016–2025	10	441.35	837.70
Quasi-Poisson	2016–2025	10	441.35	837.70
Negative binomial	2016–2025	10	441.36	837.71
ARIMAX grid search	2016–2025	10	496.87	682.06

Errors are computed on the implied annual case increment.

The raw Poisson, quasi-Poisson, and negative binomial forecasts were all below the already observed partial-year cumulative total. Since a full-year cumulative forecast cannot be smaller than the cumulative total already observed by May 1, 2026, the adjusted forecast is set to 84,565 cumulative cases for each of these models. This corresponds to 1,814 implied 2026 cases. The raw full-year ARIMAX grid-search forecast was also below the already observed partial-year cumulative total, so the same monotonicity adjustment is applied.

### 3.4 Interpretation of forecasting results

**Figure 3:**
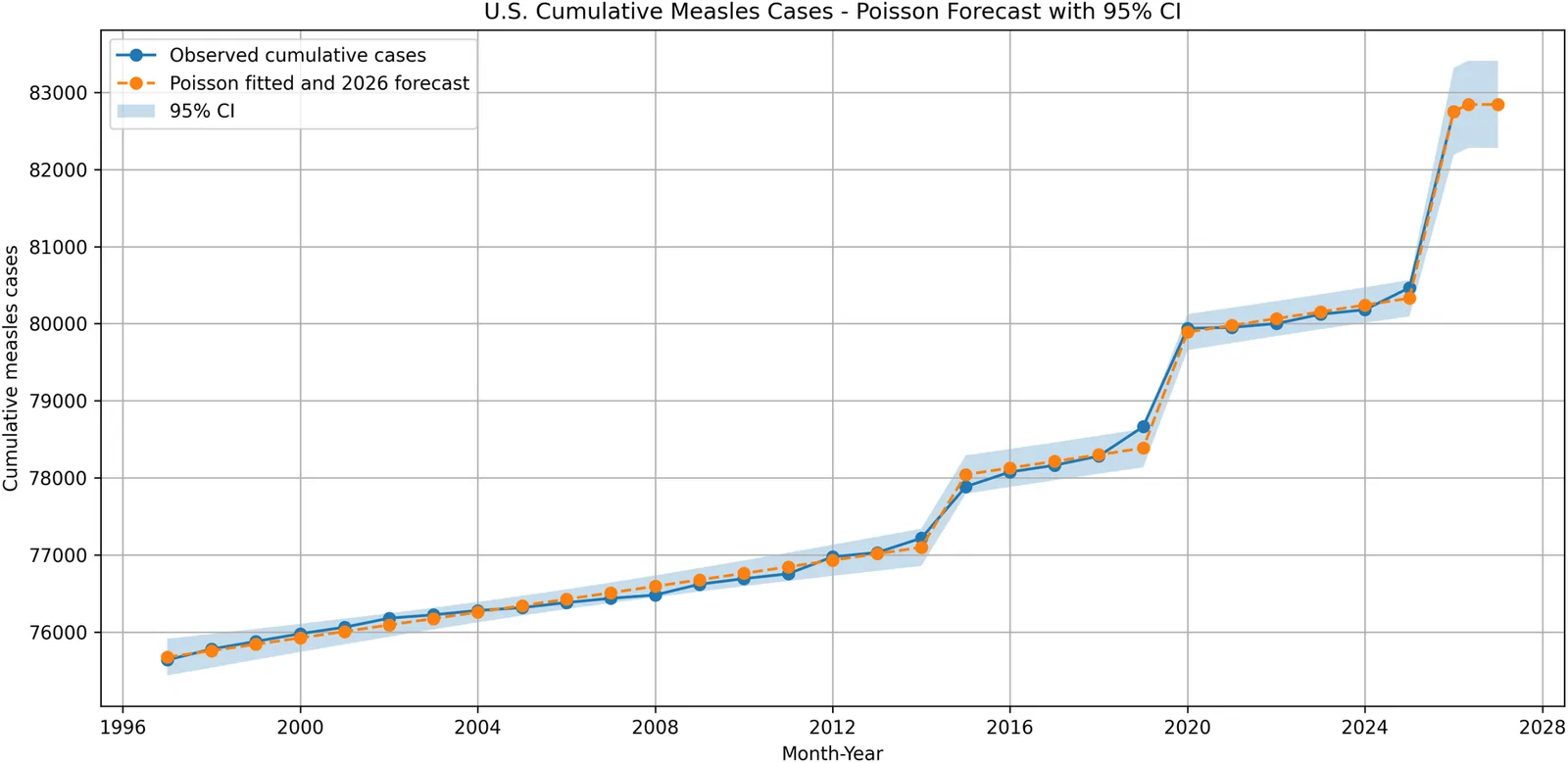
Poisson fitted cumulative cases and held-out 2026 forecast with 95% confidence interval.

**Figure 4:**
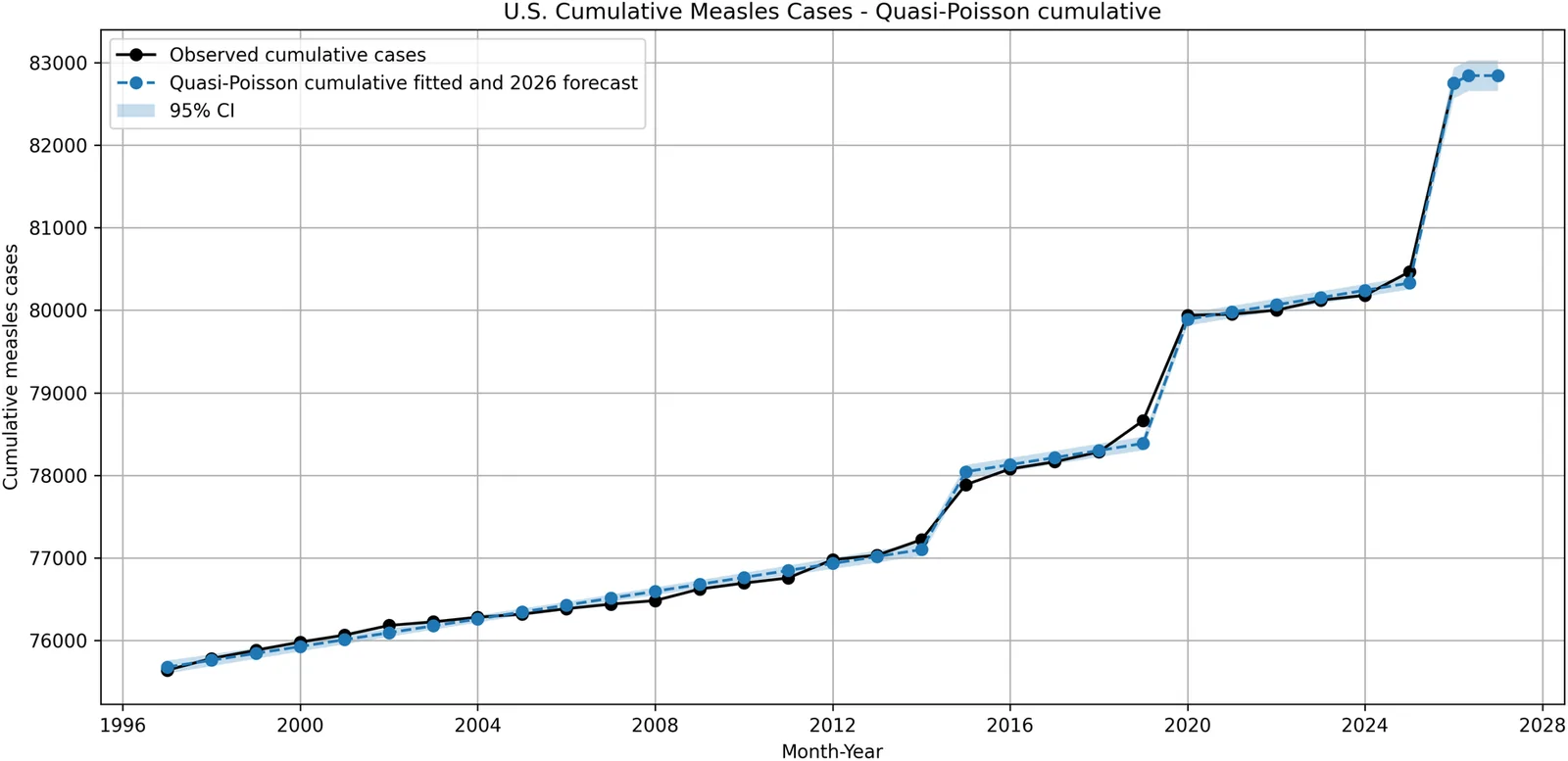
Quasi-Poisson fitted cumulative cases and held-out 2026 forecast with 95% confidence interval.

**Figure 5:**
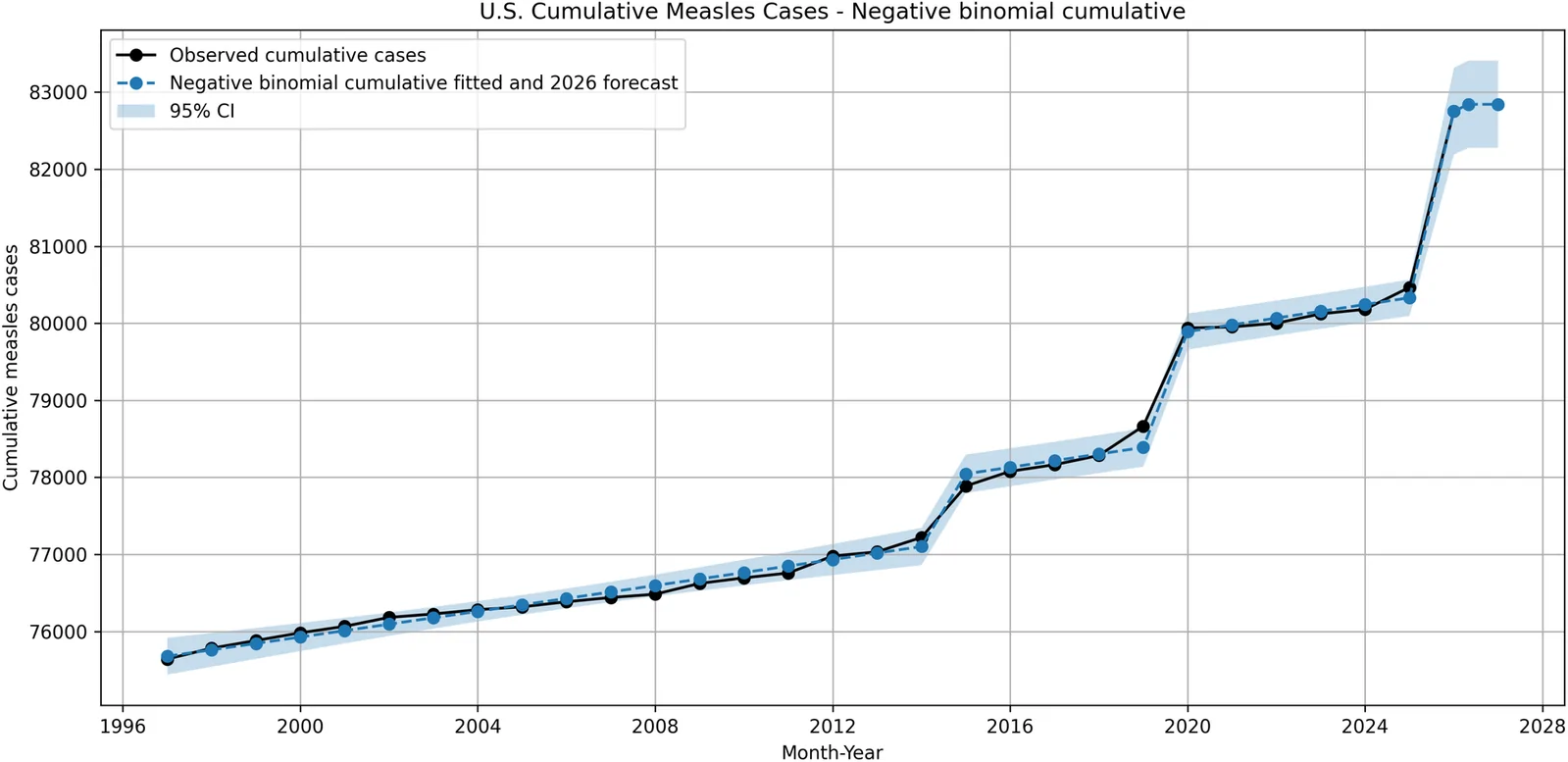
Negative binomial fitted cumulative cases and held-out 2026 forecast with 95% confidence interval.

**Figure 6:**
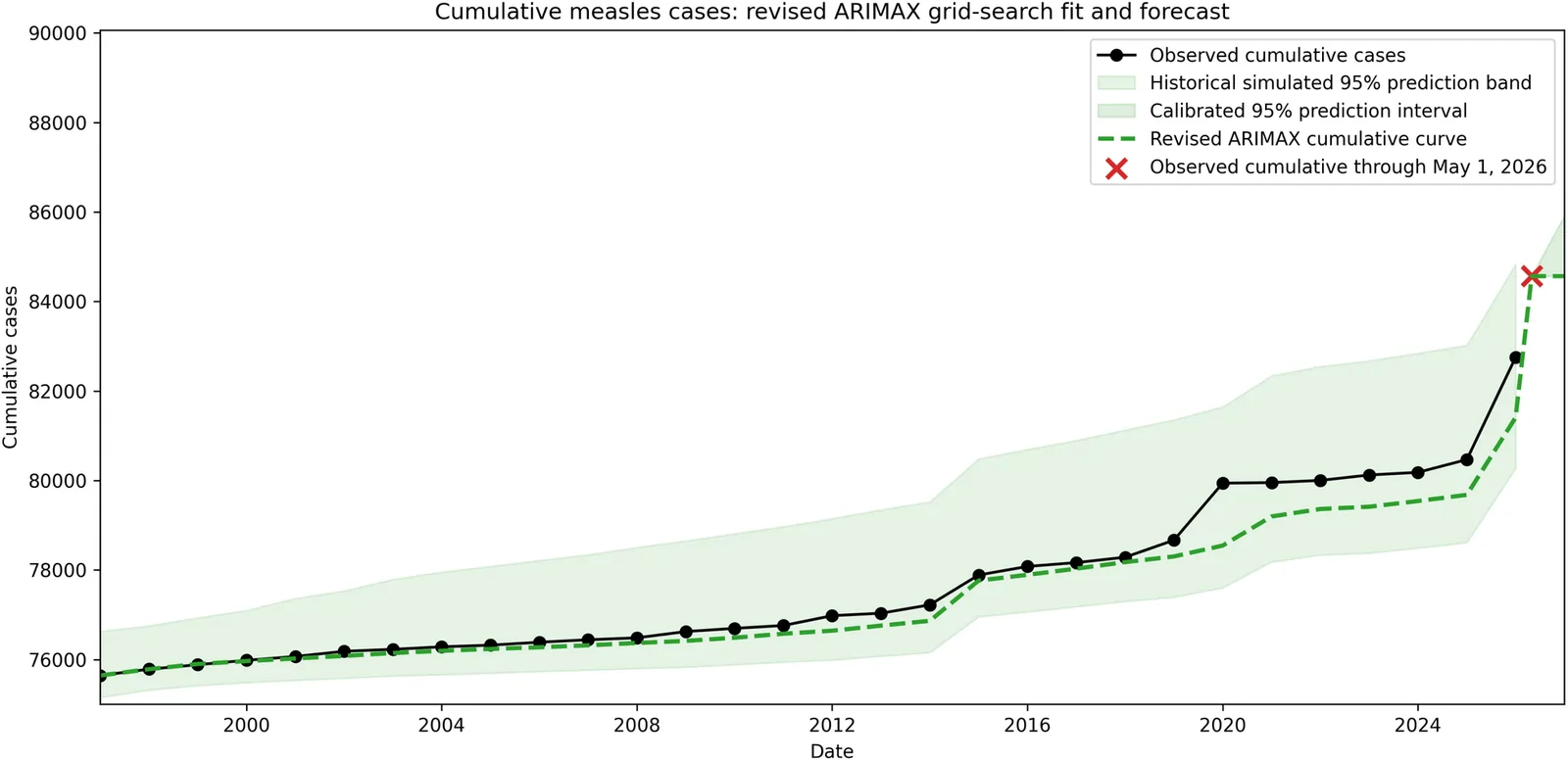
ARIMAX grid-search fitted cumulative cases and held-out 2026 forecast with 95% prediction interval.

Figures 3–6 compare the fitted cumulative measles trajectories and the held-out 2026 forecasts obtained from the Poisson GLM, quasi-Poisson GLM, negative binomial GLM, and ARIMAX grid-search models, respectively. In all figures, the fitted values closely follow the observed cumulative trajectory from 1997–2025, capturing the major upward shifts associated with the 2014, 2019, and 2025 outbreak periods.

Figure 3 shows the Poisson GLM fit and forecast. The model reproduces the historical cumulative trend with high accuracy and generates relatively narrow confidence intervals. However, the forecasted 2026 cumulative burden remains substantially below the cumulative total already observed by May 1, 2026. This indicates that the outbreak effect estimated from historical data, including the 2025 outbreak indicator, is insufficient to explain the rapid increase observed during 2026.

Figure 4 presents the quasi-Poisson model results. Because the quasi-Poisson specification retains the same mean structure as the Poisson model, the fitted trajectory and forecast are nearly identical. The primary difference lies in the variance estimation, which adjusts for potential dispersion in the data. Nevertheless, the forecast remains well below the observed partial-year cumulative burden in 2026, leading to the same substantive conclusion as the Poisson model.

Figure 5 shows the negative binomial model fit and forecast. The fitted trajectory is again almost indistinguishable from those of the Poisson and quasi-Poisson models. This result is consistent with the estimated negative binomial dispersion parameter being close to zero, causing the model to effectively collapse toward the Poisson specification. Consequently, the negative binomial forecast also underestimates the already observed 2026 cumulative burden. In contrast, Figure 6 demonstrates that the ARIMAX grid-search model produces a forecast closer to the observed cumulative total through May 1, 2026 than the count-regression models, although the raw forecast still falls short of the fully observed partial-year total. The model was fit to annual case increments on the $\log(1+C_{y})$ scale and then reconstructed into cumulative counts. Because the selected model was ARIMAX(2,0,0), the first two observations were used to initialize the autoregressive recursion. By incorporating both a one-year outbreak pulse indicator and autoregressive dependence, the ARIMAX grid-search model is able to respond more effectively to the recent upward trajectory in cumulative cases than the count-regression models, although the model exhibits weaker in-sample fit as a result. The raw 2026 cumulative forecast remained below the partial-year cumulative total already observed by May 1, 2026, so the same monotonicity adjustment described above was applied. The red marker in Figure 6 indicates the observed cumulative total through May 1, 2026, illustrating that the forecasted year-end burden was effectively reached within the first four months of the year. Taken together, the four figures provide consistent evidence that the 2026 outbreak cannot be adequately represented as a continuation of the 2025 outbreak effect alone. While the Poisson, quasi-Poisson, and negative binomial models produce very similar forecasts, all substantially underpredict the emerging 2026 burden. The ARIMAX grid-search model performs somewhat better in the held-out validation exercise but still underestimates the observed cumulative total. These findings suggest that the 2026 outbreak represents a distinct and more severe outbreak regime, warranting the introduction of a separate 2026 outbreak indicator or a broader 2025–2026 outbreak-period effect in future forecasting models.

### 3.5 Implications for future forecasting

The forecasting results indicate that the ongoing 2026 measles resurgence cannot be adequately explained solely as a continuation of the 2025 outbreak. All four models underestimated the cumulative burden observed by May 1, 2026, suggesting that recent transmission patterns differ substantially from those represented in the historical training data. Moreover, the forecasted annual burden was effectively reached within the first four months of 2026, underscoring the exceptional magnitude of the current outbreak.

These findings suggest that the 2025–2026 period may reflect transmission dynamics that are not fully captured by models calibrated using historical data alone. Consequently, future forecasting efforts should consider incorporating a dedicated outbreak indicator for 2026 or modeling the 2025–2026 period as a separate outbreak phase. Forecast accuracy may also be improved by incorporating additional explanatory variables, such as vaccination coverage, vaccine exemption rates, population mobility, vaccine hesitancy, and other epidemiologically relevant covariates. The transmission patterns observed in 2026 differ from those reported in previous years; however, these findings should be interpreted with caution. Although the observed changes may be associated with variations in population immunity, vaccination coverage, and importation events, alternative explanations, including changes in surveillance systems, testing practices, and reporting, may also have contributed to the increase in reported cases. Therefore, rather than interpreting 2026 as a definitive shift to a new epidemiological regime, we view it as a period with notable transmission characteristics that warrant further investigation using additional epidemiological data and comparative analyses with previous outbreak periods.

Across all three count-regression models, the outbreak-shift coefficients are nearly identical and statistically significant (p<0.001), corresponding to small multiplicative increases of about 1.1%, 1.8%, and 2.9% for the 2014, 2019, and 2025 shifts, respectively. The estimates agree because the quasi-Poisson model shares the Poisson mean structure and the negative binomial dispersion parameter is effectively zero, so all three models collapse to the same fit. These effects should nonetheless be interpreted cautiously against the large baseline cumulative count, which is on the order of 76,000–85,000 cases: a 2%–3% multiplicative change on that baseline still represents roughly two thousand additional cases in absolute terms, while statistical significance is easy to attain given the smoothness of the cumulative series and the correspondingly small standard errors. In the ARIMAX model, by contrast, the one-year outbreak pulse (which retains only the 2014 and 2025 pulses) is not statistically significant and has a very wide confidence interval; this imprecision reflects parameter-identifiability limitations, since only two pulses are available in a short annual series and their effect is partly absorbed by the autoregressive terms and the trend, and it is also why a separate 2019 pulse was not retained. The count-regression models are therefore the more reliable basis for quantifying outbreak magnitude, whereas the ARIMAX model is better suited to short-term prediction.

Because the outbreak effect is small on the cumulative scale in the count models and imprecisely identified in the ARIMAX model, and because all four models underpredicted the 2026 incident burden (an implied 91 additional cases under the count-regression models and 398 under the ARIMAX model, against 1,814 already observed by May 1), we base our proposed surveillance rule on the observed annual case increment rather than on any single model’s coefficient. Monitoring the cumulative total is uninformative here: the observed 2025 and 2026 cumulative totals both fell within the 95% prediction band of every model, so a cumulative interval-breach criterion would not have flagged either year. On the annual-increment scale, however, the separation is stark. Across the 1996–2024 training window, non-outbreak years averaged approximately 111 annual cases (median 86, maximum 381), whereas the three documented U.S. resurgences are far larger, at 667 cases in 2014, 1,274 in 2019, and 2,285 in 2025. An annual increment of roughly 500 cases, equivalent to about four times the non-outbreak mean and above the largest ordinary year observed, therefore separates all three resurgence years from every non-outbreak year in the training period without misclassification, and the approximately 1,814 cases already reported by May 1, 2026 exceed it more than threefold. We accordingly propose a simple, model-agnostic decision rule for prospective surveillance: introduce a new outbreak-year indicator when the observed annual incident count exceeds approximately 500 cases (about four times the recent non-outbreak average), corroborated where possible by an external public-health signal such as a CDC outbreak declaration. Because this threshold is descriptive of the annual increments and does not require estimating a pulse coefficient, it sidesteps the identifiability limitation; it is calibrated on a small number of resurgence years and should be recalibrated as additional outbreak years accrue. Thus, the comparative analysis demonstrates that accurate infectious disease forecasting requires a balance between statistical interpretability and predictive adaptability. While count-regression models provide valuable insights into the long-term effects of outbreak periods, time-series models offer greater flexibility in responding to rapidly changing transmission dynamics. Combining these strengths may provide a promising direction for future measles forecasting research and for the broader surveillance of vaccine-preventable diseases.

## 4 Discussion and conclusions

### 4.1 Discussions

This study compared four statistical frameworks for forecasting cumulative measles burden in the United States: a Poisson generalized linear model (GLM), a quasi-Poisson GLM, a negative binomial GLM, and an ARIMAX grid-search model. Collectively, the results demonstrate that each framework captures different characteristics of the surveillance data and highlight the importance of selecting models that are consistent with both the count nature of infectious disease outcomes and the temporal dependence inherent in epidemiological time series.

The Poisson GLM provided a compact and interpretable description of the cumulative measles trajectory. The log-link formulation ensured positive fitted values, while exponentiated coefficients quantified multiplicative changes in cumulative disease burden. As shown in Figure 3, the model closely tracked the historical cumulative trajectory and achieved strong in-sample performance, with training MAE (=64.60) and RMSE (=83.85). The positive outbreak indicators associated with the 2014, 2019, and 2025 resurgence periods suggest persistent upward shifts in cumulative burden, which is epidemiologically plausible because cumulative counts permanently retain the effects of major outbreaks.

The quasi-Poisson and negative binomial models were introduced to assess whether departures from the standard Poisson variance assumption materially affected model performance. As illustrated in Figures 4, 5, both models produced fitted trajectories and forecasts that were nearly indistinguishable from those of the Poisson model. The quasi-Poisson model retained the same mean structure as the Poisson model, while the estimated negative binomial dispersion parameter was effectively zero and statistically insignificant, causing the negative binomial model to converge toward the Poisson specification. Consequently, all three count-regression models yielded nearly identical forecasts and in-sample accuracy measures.

The ARIMAX grid-search model provided a complementary time-series perspective by combining a one-year outbreak-pulse indicator with autoregressive dependence. The selected ARIMAX(2,0,0) specification indicates that recent annual measles activity contains valuable information for short-term forecasting. Although the ARIMAX grid-search model exhibited weaker in-sample performance than the count-regression models, with training MAE = 341.24 and RMSE = 499.82, it generated a more accurate held-out forecast for 2026 than the count-regression models. As shown in Figure 6, the forecasted cumulative burden of 83,148.71 cases was approximately 1,416.29 cases below the observed cumulative total of 84,565 reported through May 1, 2026, a smaller gap than that of the count-regression models.

The held-out 2026 validation exercise provides the most important insight of this study. The Poisson, quasi-Poisson, and negative binomial models forecast approximately (82,842) cumulative cases for 2026, underestimating the cumulative total already observed by May 1, 2026 by approximately (1,723) cases. Although the ARIMAX grid-search model reduced this forecasting error, it also underpredicted the observed cumulative burden. The fact that all four models underestimated the already observed 2026 burden indicates that the current outbreak cannot be adequately represented as a continuation of the 2025 outbreak effect alone. Because this comparison relies on a single partial-year 2026 observation, we supplemented it with rolling one-year-ahead validation over the 2016–2025 test period for all four models. This validation refits each model using only data available before each test year and then evaluates the implied annual case increment for that year, thereby preserving the temporal ordering required for forecast assessment.

This finding suggests the presence of a structural shift in the recent epidemiological dynamics of measles transmission in the United States. The outbreak indicators included in the models successfully captured the effects of previous resurgence periods; however, the rapid growth observed during 2026 exceeds what would be expected based solely on historical outbreak patterns. Moreover, the forecasted annual burden was effectively reached within the first four months of the year, implying that the final 2026 cumulative burden will likely surpass the 2025 outbreak by a substantial margin.

From a public health perspective, these findings reinforce concerns regarding declining vaccination coverage, vaccine hesitancy, imported infections, and the accumulation of susceptible individuals within under-vaccinated communities. The persistence of outbreak-related effects in the cumulative series demonstrates how relatively short periods of elevated transmission can have long-term impacts on national disease burden. Consequently, forecasting frameworks that account for both count-data characteristics and temporal dependence can provide valuable support for surveillance, preparedness planning, and resource allocation.

Therefore, the comparison reveals a tradeoff between in-sample fit and short-term predictive performance. The count-regression models provide superior historical fit and straightforward epidemiological interpretation, whereas the ARIMAX grid-search model more effectively captures recent temporal dynamics and produces more accurate short-term forecasts. Nevertheless, all four approaches lead to the same substantive conclusion: the 2026 outbreak appears to represent a distinct and more severe outbreak regime than that captured by the 2025 outbreak effect. As additional surveillance data become available, future models should incorporate a dedicated 2026 outbreak indicator or explicitly model the 2025–2026 period as a broader outbreak regime before generating forecasts for 2027 and beyond.

### 4.2 Insights from model comparison and differences

A central contribution of this study is the comparative evaluation of four forecasting frameworks for cumulative measles burden in the United States: the Poisson GLM, quasi-Poisson GLM, negative binomial GLM, and ARIMAX grid-search model. While all four approaches were able to capture the long-term growth pattern of cumulative measles cases, important differences emerged in their forecasting behavior, predictive performance, and ability to adapt to the evolving 2026 outbreak. These differences provide valuable insights into the strengths and limitations of count-regression and time-series approaches for infectious disease forecasting.

The Poisson, quasi-Poisson, and negative binomial models performed exceptionally well in reproducing the historical cumulative trajectory. Because cumulative measles counts increase monotonically over time and retain the effects of previous outbreaks, the inclusion of outbreak-related covariates allowed these models to capture the major upward shifts associated with the 2014, 2019, and 2025 resurgence periods. As a result, all three count-regression models achieved excellent in-sample performance and generated nearly identical fitted trajectories. The similarity of these results is not surprising because the quasi-Poisson model shares the same mean structure as the Poisson model, while the estimated negative binomial dispersion parameter was effectively zero, causing the negative binomial model to converge toward the Poisson specification.

Despite their strong historical fit, the count-regression models systematically underpredicted the cumulative burden already observed during 2026. This result highlights an important limitation of regression-based forecasting approaches that rely heavily on historically estimated outbreak effects. Since the models were trained using outbreak indicators representing previous resurgence periods, they implicitly assumed that future outbreaks would behave similarly to those observed in the past. The emerging 2026 outbreak appears to violate this assumption, suggesting the presence of a new outbreak regime that is not adequately captured by the existing outbreak covariates.

In contrast, the ARIMAX grid-search model provided a more accurate held-out forecast than the count-regression models despite exhibiting weaker in-sample performance. This apparent paradox can be explained by the different sources of information used by the models. Whereas the count-regression approaches rely primarily on outbreak indicators and long-term trends, the ARIMAX grid-search model explicitly incorporates serial dependence through autoregressive terms in addition to a one-year outbreak-pulse indicator. Consequently, the model is able to utilize recent changes in the annual case trajectory and respond more effectively to emerging outbreak dynamics.

The relative advantage of the ARIMAX grid-search model for short-term prediction is particularly evident in the 2026 validation exercise. The count-regression models forecast approximately 82,842 cumulative cases, implying only a modest increase beyond the 2025 total. In contrast, the ARIMAX grid-search model forecast 83,148.71 cumulative cases, closer to the observed cumulative total of 84,565 cases reported through May 1, 2026. This result suggests that recent transmission dynamics contained predictive information that could not be fully represented by outbreak indicators alone. Although the ARIMAX grid-search model also underpredicted the observed burden, its smaller forecasting error demonstrates the value of incorporating temporal dependence when forecasting rapidly evolving outbreaks.

Taken together, these findings reveal an important tradeoff between explanatory performance and predictive performance. The count-regression models provide superior interpretability and historical fit because their parameters directly quantify the effects of major outbreak periods on cumulative disease burden. However, these models may be less responsive to sudden structural changes that differ from previously observed outbreaks. Conversely, the ARIMAX grid-search model sacrifices some interpretability and in-sample accuracy but gains the ability to adapt to recent trends and improve short-term forecasting performance. Therefore, the choice of forecasting framework depends on the primary objective of the analysis, whether explanation of historical disease dynamics or prediction of future outbreak behavior.

### 4.3 Model strengths and limitations

A major strength of this study is the systematic comparison of multiple statistical frameworks for forecasting cumulative measles burden within a unified analytical setting. By evaluating Poisson, quasi-Poisson, negative binomial, and ARIMAX grid-search models using the same surveillance dataset and outbreak indicators, the analysis provides a comprehensive assessment of both count-regression and time-series forecasting approaches. The count-regression models offer several advantages. The Poisson GLM provides a simple and highly interpretable framework in which model coefficients have direct multiplicative interpretations. The quasi-Poisson model relaxes the strict Poisson variance assumption while preserving the same mean structure, allowing the sensitivity of inference to dispersion assumptions to be evaluated. The negative binomial model provides a flexible alternative capable of accommodating overdispersion when present. In this study, however, the estimated negative binomial dispersion parameter was effectively zero, resulting in fitted values and forecasts that were nearly identical to those of the Poisson and quasi-Poisson models. The ARIMAX grid-search model complements the count-regression approaches by explicitly accounting for serial dependence in the annual measles series. Although its in-sample fit was weaker than that of the count-regression models, it produced a more accurate held-out forecast for 2026, demonstrating the value of incorporating temporal dependence when forecasting emerging outbreak activity. Several limitations should be acknowledged. First, the analysis relies on annual aggregated surveillance data, which may mask seasonal transmission patterns and within-year outbreak dynamics. Second, the models utilize outbreak indicators as explanatory variables but do not explicitly incorporate potentially important drivers of measles transmission such as vaccination coverage, exemption rates, population mobility, demographic characteristics, healthcare access, or socioeconomic conditions. Third, the relatively small number of annual observations limits the complexity of models that can be estimated reliably and reduces the statistical power available for model selection and inference, as reflected in the wide confidence interval around the outbreak-pulse coefficient in the ARIMAX model. Fourth, the primary out-of-sample validation relies on a single partial-year 2026 observation, which by itself provides limited evidence of forecasting performance; we addressed this limitation by supplementing it with rolling one-year-ahead validation over the 2016–2025 test period for all four models. Finally, because 2026 was intentionally withheld for validation, no dedicated 2026 outbreak indicator could be estimated, which likely contributed to the systematic underprediction observed across all forecasting approaches.

### 4.4 Comparison with existing studies

Previous studies of measles dynamics have focused primarily on mechanistic compartmental models, outbreak investigations, vaccination policy analyses, and assessments of herd immunity. These studies have provided important insights into transmission dynamics, vaccine effectiveness, and the factors contributing to measles resurgence. However, relatively few investigations have focused on statistical forecasting of cumulative measles burden in the post-elimination era of the United States.

The findings of this study are consistent with a broader literature demonstrating that infectious disease surveillance data often exhibit temporal dependence and structural changes associated with outbreak events. Similar observations have been reported for influenza, dengue fever, COVID-19, and other communicable diseases, where both regression-based and time-series approaches have been used to improve forecasting performance. Additionally, recent advances in infectious disease forecasting have increasingly combined classical statistical methods with artificial intelligence and machine learning techniques to improve predictive performance and support public health decision-making. For example, Baker et al. (19) developed a comprehensive forecasting framework that compared ARIMA, Prophet, and LSTM models using COVID-19 data from New Zealand and its partner countries, demonstrating that adaptive, data-driven forecasting systems can provide valuable early warning information for epidemic preparedness. Similarly, Baker et al. (20) proposed a collaborative machine learning framework for disease prediction that highlights the benefits of integrating complementary predictive models to enhance forecasting accuracy and reliability. These recent studies underscore the growing importance of robust forecasting methodologies for infectious diseases and provide further motivation for evaluating multiple statistical and time-series models in the present study.

This work contributes to the existing literature by directly comparing count-regression and time-series forecasting approaches while explicitly incorporating outbreak-related structural shifts into cumulative burden models. Unlike studies that focus solely on annual incidence, the present analysis emphasizes cumulative disease burden, thereby capturing the persistent impact of major outbreaks on long-term national trends. The results further demonstrate that outbreak indicators alone may be insufficient when a new outbreak regime emerges, as evidenced by the systematic underprediction of the 2026 burden across all models.

### 4.5 Concluding remarks

This study investigated the forecasting of cumulative measles burden in the United States using Poisson, quasi-Poisson, negative binomial, and ARIMAX grid-search models. The results demonstrate that each framework captures different aspects of the surveillance data and that no single model is uniformly superior across all performance criteria. The Poisson, quasi-Poisson, and negative binomial models provided excellent in-sample fit and yielded nearly identical fitted trajectories and forecasts. In contrast, the ARIMAX grid-search model incorporated temporal dependence and produced a more accurate held-out forecast for the 2026 validation period. Nevertheless, all four models underestimated the cumulative burden already observed by May 1, 2026, indicating that the ongoing outbreak cannot be adequately represented as a continuation of the 2025 outbreak effect alone. The forecasting results suggest that the 2026 resurgence represents a distinct and potentially more severe outbreak regime than those observed in recent years. The fact that the forecasted annual burden was effectively reached within the first four months of 2026 highlights the magnitude of the current outbreak. It underscores the limitations of relying solely on historical outbreak patterns for prediction. Thus, the findings emphasize the importance of combining epidemiological insight with statistical forecasting methods when assessing future disease burden. As additional data become available, models that explicitly account for the 2026 outbreak and incorporate relevant epidemiological drivers will likely provide more reliable forecasts and stronger support for public-health planning, vaccination policy development, and outbreak preparedness efforts in the United States.

Several directions for future research emerge from this study. First, as additional surveillance data become available, the forecasting models should be re-estimated to incorporate a dedicated 2026 outbreak indicator and quantify the persistence of the current resurgence. Such analyses may improve forecasting accuracy and provide insight into whether the 2026 outbreak represents a temporary surge or a longer-term structural shift in measles transmission dynamics. Second, future studies should investigate count time-series methodologies specifically designed for epidemiological data, including generalized autoregressive moving average models, integer-valued autoregressive processes, dynamic negative binomial models, and state-space count models. These approaches may improve predictive performance while preserving the discrete nature of disease counts.

Finally, extending the analysis to state-level or regional surveillance data would permit investigation of spatial heterogeneity in outbreak risk. Spatial and spatiotemporal modeling approaches could provide additional insight into localized transmission dynamics and support geographically targeted public-health interventions.

## Data Availability

The original contributions presented in the study are included in the article/Supplementary Material, further inquiries can be directed to the corresponding author.

## References

[1] Centers for Disease Control and Prevention. Measles-United States, 2000. MMWR Morb Mortal Wkly Rep. (2002) 51:120–3. Available online at: https://www.cdc.gov/mmwr/preview/mmwrhtml/mm5106a2.htm 11898926

[2] Fiebelkorn AP Redd SB Gallagher K Rota PA Rota J Bellini W, et al. Measles in the United States during the postelimination era. J Infect Dis. (2010) 202:1520–8. 10.1086/656914 20929352

[3] McCullagh P Nelder JA. Generalized Linear Models. 2nd edn. Chapman and Hall (1989).

[4] Hilbe JM. Negative Binomial Regression. Vol. 2. New York, NY: Cambridge University Press (2011).

[5] Farrington CP Andrews NJ Beale AD Catchpole MA. A statistical algorithm for the early detection of outbreaks of infectious disease. J R Stat Soc Ser A. (1996) 159:547–63. 10.2307/2983331

[6] Held L Höhle M Hofmann M. A statistical framework for the analysis of multivariate infectious disease surveillance counts. Stat Modell. (2005) 5:187–99. 10.1191/1471082X05st098oa

[7] Liboschik T Fokianos K Fried R. tscount: an R package for analysis of count time series following generalized linear models. J Stat Softw. (2017) 82:1–51. 10.18637/jss.v082.i05

[8] Anderson RM May RM. Infectious Diseases of Humans: Dynamics and Control. Oxford University Press (1991).

[9] Patel M Lee AD Clemmons NS Redd SB Poser S Blog D. National update on measles cases and outbreaks-United States, January 1–October 1, 2019. MMWR Morb Mortal Wkly Rep. (2019) 68:893–6. 10.15585/mmwr.mm6840e2 31600181 PMC6788396

[10] Mathis AD Raines K Filardo TD Wiley N Leung J Rota PA. Measles update-United States, January 1-April 17, 2025. MMWR Morb Mortal Wkly Rep. (2025) 74:233–9. 10.15585/mmwr.mm7414a1 PMC12021071 40273019

[11] Ojasanya R Awosile BB Adeyemo P Jarra E Berke O. Time series analysis of measles incidence in Nigeria using surveillance data from 2011 to 2022. McGill J Glob Health. (2025) 14:23–7. 10.26443/mjgh.v14i1.1548

[12] Box GE Jenkins GM Reinsel GC Ljung GM. Time Series Analysis: Forecasting and Control. 5th edn. John Wiley & Sons (2015).

[13] Hyndman RJ Khandakar Y. Automatic time series forecasting: the forecast package for R. J Stat Softw. (2008) 27:1–22. 10.18637/jss.v027.i03

[14] Hyndman RJ Athanasopoulos G. Forecasting: Principles and Practice. 3rd edn. OTexts (2021).

[15] Chukwu CW. Forecasting and quantifying the role of vaccination strategies in the 2025 Texas measles outbreak: a modeling and time series analysis approach. BMC Infect Dis. (2026) 26:530. 10.1186/s12879-026-12720-0 41654706 PMC12977517

[16] Centers for Disease Control and Prevention. Data from: Measles cases and outbreaks (2026). Available online at: https://www.cdc.gov/measles/data-research/index.html (Accessed July 11, 2026).

[17] Beacon Bio. Data from: Measles surveillance report (2026). Available online at: https://beaconbio.org/en/report/?reportid=620a7dc7-3793-4754-bd78-28c7fb6aa816&eventid=2f15d768-f7ee-48ec-b675-02ad2862880f (Accessed July 15, 2026).

[18] Chukwu CW Obaido G Mienye ID Aruleba K Esenogho E Modisane C. Time series modeling of monkeypox incidence in Central Africa’s endemic regions. Mach Learn Appl. (2025) 22:100778. 10.1016/j.mlwa.2025.100778

[19] Baker O Ziran Z Mecella M Subaramaniam K Palaniappan S. Predictive modeling for pandemic forecasting: a COVID-19 study in New Zealand and partner countries. Int J Environ Res Public Health. (2025) 22:562. 10.3390/ijerph22040562 40283787 PMC12026713

[20] Baker O Subaramaniam K Shibghatullah ASB Shaffiei ZA Hamzah ASSSA. A collaborative framework for disease prediction using machine learning. In: *2025 IEEE international conference on artificial intelligence in engineering and technology (IICAIET)* (IEEE) (2025), 650–654. 10.1109/IICAIET67254.2025.11265309

